# Reply to “Inconsistencies in the specific nucleobase pairing motif prone to photodimerization in a MOF nanoreactor”

**DOI:** 10.1038/s41467-022-30193-y

**Published:** 2022-08-02

**Authors:** Samantha L. Anderson, Peter G. Boyd, Andrzej Gładysiak, Tu N. Nguyen, Robert G. Palgrave, Dominik Kubicki, Lyndon Emsley, Darren Bradshaw, Matthew J. Rosseinsky, Berend Smit, Kyriakos C. Stylianou

**Affiliations:** 1 Laboratory of Molecular Simulation (LSMO), Institut des Sciences et Ingénierie Chimiques (ISIC), École Polytechnique Fédérale de Lausanne (EPFL), Rue de l’Industrie 17, CH-1951 Sion, Switzerland; 2 University College London, Department of Chemistry, 20 Gordon St, London, WC1H 0AJ UK; 3 Laboratory of Magnetic Resonance (LRM), Institut des Sciences et Ingénierie Chimiques (ISIC), École Polytechnique Fédérale de Lausanne (EPFL), CH-1015 Lausanne, Switzerland; 4 School of Chemistry, University of Southampton, Highfield Campus, Southampton, UK; 5 Department of Chemistry, University of Liverpool, Crown Street, Liverpool, L69 7ZD UK; 6 Department of Chemistry, Oregon State University, Corvallis, OR 97331 USA

**Keywords:** Metal-organic frameworks, Supramolecular chemistry

**replying to** Clivio et al. *Nature Communications* 10.1038/s41467-021-27196-6 (2021)

In response to Dr. Clivio’s comments.

Dr. Clivio’s comments correctly point out that the choice of a particular stereoisomer shown as an illustration in Fig. 4c in our original manuscript^1^ was unfortunate, as we selected an isomer that turns out to be inconsistent with what the experimental data suggested. We had carried out some preliminary density functional theory (DFT) calculations to investigate the formation of the four different stereoisomers within the MOF pores (see Fig. 1 below). These calculations showed that the **trans-syn (A)** has the lowest energy (−24.5 kcal/mol), while the other isomers have energy in the range −10 to 13 kcal/mol, suggesting that experimentally one might preferentially form the **trans-syn (A)**. These DFT calculations were performed at 0 K, while the experiments were done at room temperature. The minimum energy conformation of adenine-thymine bonding at 0 K would provide us with limited information about the behavior of thymine in the pores of the MOF at room temperature, which is why we turned to molecular dynamic simulations (MD) at room temperature. The results shown in Fig. 3c,d in our original manuscript^1^ are from MD simulations. These MD simulations were not restricted to one particular isomer. We agree with Dr. Clivio that if we would have limited our calculations to only the structure shown in Fig. 4c in the original manuscript^1^ would have introduced a bias, but in our MD simulations, we did not.

**Fig. 1:**
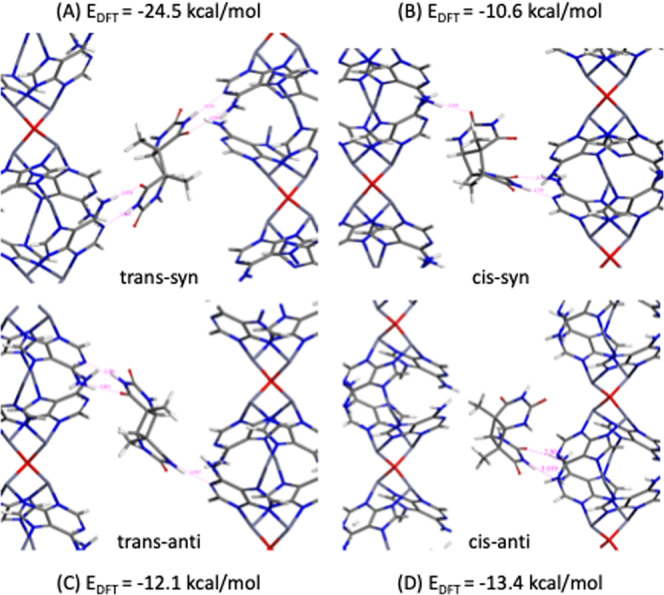
The four possible Thy<>Thy isomers that can form in the pores of the MOF. **A**–**D** The DFT energies for the different stereoisomers at 0 K.

We concluded that the experimental data, while able to confirm the dimer formation, were unable to define whether a specific stereoisomer was favored. We, therefore, selected one stereoisomer for illustration, ***trans-anti (C)***, without any particular purpose in Fig. 4c. However, we do want to point out the following: in Dr. Clivio’s comment, the author mentioned, “ ….the authors provide evidence (see Fig. 3c,d, 4 in ref. ^1^.) clearly indicating that the photodimer is ***trans-anti***, …” This is factually incorrect, as nowhere in our manuscript^1^, we make this claim. In fact, we incorrectly concluded that our experimental data did not provide any indication of which isomer would form. Given this conclusion, we used a random isomer for illustration purposes, but nowhere in the article we mention explicitly or implicitly that this photodimer is preferentially formed.

We are delighted to see that Dr. Clivio’s comment points out that our UHPLC- ESI/MS spectra of the SION-19-derived photoproduct show a fragment ion at m/z 210 (see Supplementary Figs. 36–37 and 41 in our original manuscript) which is characteristic of a **syn (A) or (B)** thymine dimer isomer^2,3^. This does not yet confirm our prediction, but it is an encouraging result as it excludes the formation of **anti (C) or (D)**.

Finally, we do agree with Dr. Clivio that further HPLC experiments would be an interesting next step to further explore the stereoisomers present, and we also welcome additional suggestions to rigorously determine the specific mode of binding between host (MOF) and guest (thymine) at the supramolecular level and the stereochemistry of the dimerized photoproduct of these very interesting systems.

## Data Availability

All data supporting the findings of this study are available from the original publication or from the authors upon reasonable request.
